# Roles and mechanisms of *gapA* and *gap*-encoded proteins in *cronobacter sakazakii* adhesion to and invasion of intestinal cells and neonatal rats

**DOI:** 10.1080/21505594.2024.2446713

**Published:** 2024-12-30

**Authors:** Chumin Zhao, Ping Li, Dongdong Zhu, Zehui Xiao, Jingbo Jiao, Yue Li, Xinjun Du, Shuo Wang

**Affiliations:** a State Key Laboratory of Food Nutrition and Safety, College of Food Science and Engineering, Tianjin University of Science and Technology, Tianjin, China; b Tianjin Key Laboratory of Food Science and Health, School of Medicine, Nankai University, Tianjin, China

**Keywords:** *Cronobacter sakazakii*, adhesion, GAPDH, *gapA*, *gap*, neonatal rat

## Abstract

*Cronobacter sakazakii* (*C. sakazakii*) is a foodborne pathogen whose initial process involves intestinal cell adhesion mediated by numerous virulence factors encoded in various genes. The key metabolic enzyme, glyceraldehyde-3-phosphate dehydrogenase (GAPDH, also known as GapA), is encoded by *gapA* in the glycolysis pathway and acts as an adhesin in some bacteria. In *C. sakazakii*, there is also a key enzyme in the gluconeogenesis pathway, Gap, with type I GAPDH and erythrose-4-phosphate dehydrogenase activities and encoded by *gap*. This study aimed to investigate the virulence properties of GapA and Gap of *C. sakazakii* in adhesion to and invasion of HCT-8 and HIEC-6 cells and damage to the colon and brain of neonatal rats, by gene silencing. In addition, the role of both recombinant proteins in intestinal cell adhesion and invasion was investigated, and their role in inducting inflammatory cytokine expression was assessed by ELISA and Western blot. Silencing *gapA* or *gap* decreased the viability and swimming motility of bacterial cells and reduced bacterial adhesion to and invasion of both types of intestinal cells. Both recombinant proteins contributed to *C. sakazakii* adhesion in both cells, enhanced protein phosphorylation of NF-κB, and induced inflammatory cytokine expression. Finally, silenced expression of GapA and Gap also weakened bacterial damage to the brain and colon of neonatal rats. In conclusion, we demonstrated for the first time the virulence properties of GapA and Gap in *C. sakazakii* adhesion to and invasion of intestinal cells and neonatal rats and induction of inflammatory responses.

## GRAPHICAL ABSTRACT

**Figure:**
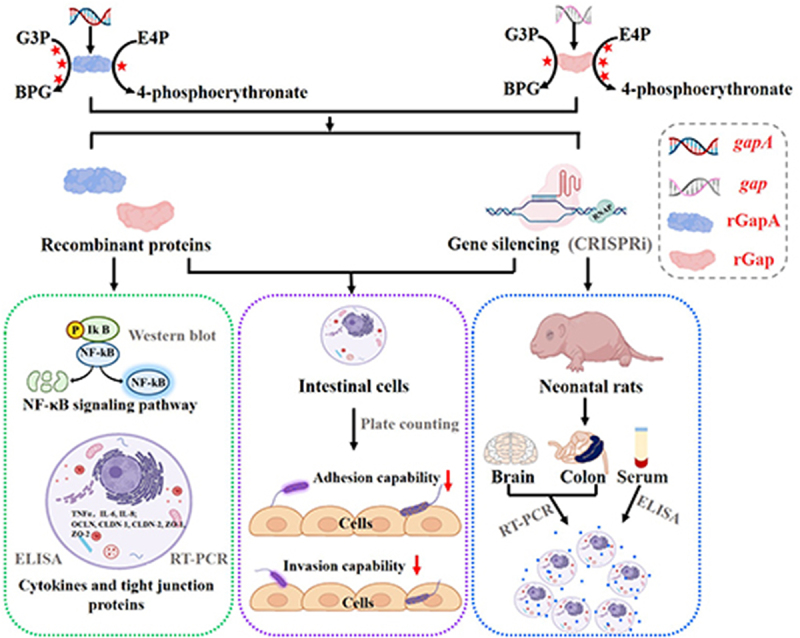

## Introduction

Most pathogens can adhere to the surface of intestinal cells through protein-host interactions, eventually leading to systemic infection. The intestinal barrier is one of the first and most important defence mechanisms against the invasion of external pathogens, but an increasing number of pathogens have gradually developed multiple strategies to disrupt the intestinal barrier and the host immune system [1]. Several extracellular proteins known as adhesins can induce host immune responses and assist in bacterial adhesion and colonization. Adhesins include pili and non-pilus adhesins, including lectins, type III secretion system proteins, and proteins based on glycoprotein interaction [2].

Moonlighting proteins are proteins that are involved in both essential cellular processes such as glycolysis and exhibit additional functions such as adhesion due to their multiple localizations in the cytoplasm, cell surface, and extracellular, such as glyceraldehyde-3-phosphate dehydrogenase (GAPDH) in the glycolysis pathway [3]. It has been shown that GAPDH can as an adhesin contribute to some bacterial adhesion to and colonization of intestinal cells, and in particular GAPDH can act as a receptor for plasminogen to induce extracellular matrix degradation, which further enhances bacterial adherence to intestinal cells and leads to systemic infection [4–6]. Multiple copies of GAPDH with different physiological functions and subcellular localizations have been identified in a variety of eukaryotes and plants, such as TDH1, TDH2, and TDH3 in *Saccharomyces cerevisiae*, and two functionally opposite types of GAPDH have been found to co-exist in bacterial cells such as *Bacillus subtilis*, *Neisseria meningitidis*, *Neisseria gonorrhoeae*, and *Helicobacter pylori* [7].

*Cronobacter sakazakii* (*C. sakazakii*) is an opportunistic pathogen that can survive in the intestinal tract of humans and animals and cause life-threatening diseases such as necrotizing enterocolitis (NEC) and bacteraemia, mainly in neonates and infants [8]. Among clinical isolates of *C. sakazakii*, the sequence type 8 (ST8) lineages have been identified as the main pathogenic lineages and are associated with diarrhoea, including *C. sakazakii* ATCC 29544 (ST8, clonal complex 8) [8]. *C. sakazakii* initially needs to adhere to intestinal cells before invading the host, and several recognized virulence factors are involved in the initial adhesion process, including plasminogen activator Cpa, pili, outer membrane proteins A (OmpA) and X (OmpX), etc [9]. *C. sakazakii* contains two types of GAPDH, one of which is the NAD-dependent moonlighting enzyme GAPDH (also known as GapA) encoded by *gapA*, and the other is NAD^+^-dependent type I GAPDH (also known as Gap) encoded by *gap*. According to the NCBI’s Protein-Protein BLAST tool, BLASTP, Gap is present in about 45 *C. sakazakii* strains with more than 48.62% similarity to GapA of *C. sakazakii* and about 48.93–88.10% similarity to the Gap of *Escherichia coli*. The main difference between these two enzymes is that Gap is a key enzyme in the gluconeogenesis pathway with low phosphorylating GAPDH activity and efficient non-phosphorylating erythrose-4-phosphate dehydrogenase (E4PDH) activity. In contrast, GapA is a key enzyme in the glycolysis pathway with high activity of phosphorylating GAPDH and low activity of non-phosphorylating E4PDH [10]. Although GapA and Gap are localized in the cytoplasm of bacterial cells and do not contain signal peptide sequences, GapA can be detected in culture supernatants of *C. sakazakii*, and Gap may have the similar characteristic [11]. The virulence properties of Gap in bacteria have not been reported, nor have the virulence properties of GapA in *C. sakazakii*. GapA, as a potential adhesin, may play a key role in the initial adhesion process of *C. sakazakii*, while Gap, which has the opposite enzymatic activities to GapA, may also be involved in this process.

Since *gapA* is one of the housekeeping genes of *C. sakazakii* and cannot be deleted, its properties can be investigated by gene-silencing techniques such as RNA interference (RNAi) and the clustered regularly interspaced short palindromic repeats (CRISPR)/dead Cas9 (dCas9) fusion-mediated inhibition (CRISPRi) system. Among them, RNAi is limited to some organisms with specific mechanisms, and its off-target effects and toxicity cannot be ignored [12]. The CRISPR system, present in about 40% of bacteria and 90% of archaea, is a mechanism for resisting foreign DNA elements, and the CRISPRi system is developed from the type II CRISPR system of *Streptococcus pyogenes* [12,13]. The dCas9 protein, which lacks endonuclease activity, binds to DNA complementary to customizable single guide RNA (sgRNA) to block transcription, thereby silencing the target gene.

In this study, we focused on the virulence properties of GapA and Gap in *C. sakazakii* adhesion to and invasion of intestinal cells and neonatal rats, as well as their effects on inflammatory cytokine expression. In addition, the cytotoxicity of recombinant GapA and Gap, which were efficiently expressed in *E. coli*, and their role in bacteria-host interactions were investigated (see Graphical Abstract). To our knowledge, this is the first report investigating the virulence properties of GapA and Gap in *C. sakazakii*.

## Materials and methods

### Bacteria strains and plasmids

*C. sakazakii* ATCC 29544 strain grown overnight in Luria Bertani (LB) medium was inoculated into fresh LB medium at a ratio of 1:100, and bacterial cells cultured to an OD_600_ of 0.6 were collected for subsequent analysis. *E. coli* BL21 (DE3) strain and pET-26b(+) expression plasmid was used to express 6×His-tagged recombinant proteins. Gene silencing was performed using plasmid dCas9 (pdCas9, resistant to chloramphenicol) containing dCas9 nuclease and plasmid TargetF (pTargetF, resistant to spectinomycin) containing sgRNA. The oligonucleotide primers used in this study are shown in Table 1.

**Table 1.** The oligonucleotide primers used in this study.

No.	Primers	Forward (5′-3′)	Reverse (5′-3′)
For producing recombinant proteins.			
1	*gapA*	GGGAATTCCATATGACTATCAAAGTAGGTATCAACGGTTTTGGCC	CCGCTCGAGTTTGGAGATGTGAGCGATCAGGTCC
2	*gap*	GGGAATTCCATATGGTTAAAGTAGGCATTAACGGATTTGGCCG	CCGCTCGAGCAGACCGCGGCGAGCCATTAACAG
For constructing recombinant pTargetF.			
3	*gapA*-sgRNA	GGTCAACAACGGATACGTTCGTTTTAGAGCTAGAAATAGCAAGTTAAAATAAGGCTAGTCC	ACTAGTATTATACCTAGGACTGAGCTAGCTGTCAAG
4	*gap*-sgRNA	ATTCGTTATCATACCAGGCGGTTTTAGAGCTAGAAATAGCAAGTTAAAATAAGGCTAGTCC	ACTAGTATTATACCTAGGACTGAGCTAGCTGTCAAG
For validating gene silencing efficiency.			
5	*gapA*	AGCAGCAACCTACGAGCAGATCAAAG	GTTTCGTTGTCGTACCAGGAAACCAGTTTC
6	*gap*	AAGGCGATTGGTAAAGTGATCCCTGAAC	CATCGCTGTAACCAAGAATGCCTTGC
7	16S rRNA	TTACGACCAGGGCTACACACG	CGGACTACGACGCACTTTATGAG
For determining the expression of inflammatory cytokines and tight junction proteins in cells.			
8	GAPDH	GTGGCTGGCTCAGAAAAAGG	GGGGAGATTCAGTGTGGTGG
9	IL-6	AGCAGGCACCCCAGTTAATC	ATTTGTGGTTGGGTCAGGGG
10	IL-8	CTCCAAACCTTTCCACCCCA	ACTGTAATCCTAACACCTGGAAC
11	IL-1β	CTGCTGTGTCCCTAACCACAA	TAGACCTGTTCCCAGCTTTTCC
12	TNFα	TGTCTGGCACATGGAAGGTG	GCTCTTAGCCCTGAGGTGTC
13	OCLN	ATGAGACAGACTACACAACTGG	TTGTATTCATCAGCAGCAGC
14	CLDN-1	ACAAAGTCCTGCTAGTGCCA	CAGTTGAAAAGCACAGAGCTTGA
15	CLDN-2	ATCCTTTATCACCTCAGCCCGT	GGGAACAAATACCACCAACCC
16	ZO-1	ACCAGTAAGTCGTCCTGATCC	TCGGCCAAATCTTCTCACTCC
17	ZO-2	GCCGCTAAGAGCACAGCAA	TCCCCACTCTGAAAATGAGGA
For determining the expression of inflammatory cytokines and tight junction proteins in brains and colons.			
18	GAPDH	ACCATCTTCCAGGAGCGAGATC	GTGGTTCACACCCATCACAAACATG
19	IL-6	AGAGGAGACTTCACAGAGGATACCAC	CATCATCGCTGTTCATACAATCAGAATTGCC
20	IL-10	AGAAGGACCAGCTGGACAACATAC	GATTTCTGGGCCATGGTTCTCTG
21	IL-1β	CTCACAGCAGCATCTCGACAAG	GGTCGTCATCATCCCACGAGTC
22	TNFα	AGACCCTCACACTCAGATCATCTTCTC	GTAGATAAGGTACAGCCCATCTGCTG
23	INFγ	GGATATCTGGAGGAACTGGCAAAAGG	CTGTTGCTGAAGAAGTTAGTGATCAGGTG
24	OCLN	ACAAGTGGACGTCGCCTC	CGTAGCCGTAACCGTAACCTC
25	CLDN-1	ACAACATCGTGACTGCTCAGG	CCAATTACCATCAAGGCTCTGGTTG
26	CLDN-2	ACTGGCATCACCCAGTGTGATATC	CTGGCAGAACACTGTGCATCTC
27	ZO-1	CAGATAGTATCCATTCTGCTAATGCCTCTG	TGCTGTGGAGACTGTGTGGAATG
28	ZO-2	AGTGGTAGACACGCTGTACGATG	CGCCAGAAGTCTGCTCTGTC

### Phylogenetic tree

Construction of phylogenetic trees based on amino acid sequences of GapA and Gap proteins in different organisms. The amino acid sequences of GapA and Gap of *C. sakazakii* ATCC 29544 were set as reference sequences, respectively, and the BLASTP tool was used to search for the amino acid sequences of GapA or Gap from a total of 39 organisms in five different categories of organisms, including animals, plants, fungi, protists, and bacteria. The amino acid sequences were aligned using the ClustalW tool in MEGA-X software v.10.1.8 with default parameters, followed by construction of phylogenetic trees using MEGA-X and the neighbour-joining (NJ) method with 1000 bootstrap replicates [14]. The visualization of the phylogenetic tree was performed using the Interactive Tree of Life webserver [15].

### Production of recombinant proteins

*gapA* and *gap* with the addition of *Nde*I and *Xho*I restriction sites were amplified by the polymerase-chain reaction (PCR). pET-26b(+) and amplified DNA were digested by *Nde*I and *Xho*I restriction enzymes (Takara, Japan) and ligated by T4 DNA ligase (Thermo Fisher Scientific, USA), followed by verification of insertion sequences in the recombinant plasmids by sequencing and transformation of recombinant plasmids into *E. coli* BL21 (DE3) chemically competent cells using the heat shock method. The production of recombinant proteins was induced by 0.5 mm Isopropyl β-D-Thiogalactoside for 8 h at 25°C. Bacterial cells were broken using a high-pressure cell crusher (Constant Systems, UK), followed by centrifugation for 15 min at 12,400 × g to collect the supernatant. The supernatant was then applied to a High-Affinity Ni-Charged Resin FF column (Genscript, China), and the recombinant proteins were eluted with different concentrations of imidazole under native conditions. The production efficiency of recombinant proteins was verified by 12% sodium dodecyl sulphate-poly acrylamide gel electrophoresis (SDS-PAGE) gels, and imidazole was removed by dialysis with PB buffer (25.22 mm Na₂HPO_4_ and 13.00 mm NaH_2_PO_4_). The recombinant proteins were concentrated and quantified using the Centrifugal Filter Device (Ultra-15, 10000 NMWL, Millipore, USA) and the Coomassie Blue Fast Staining Solution (Beyotime Biotechnology, China), respectively [16,17].

### Production of polyclonal antibodies

Polyclonal antibodies were prepared according to the previous study [18]. Rabbits (New Zealand White) were immunized with 2 mg of recombinant GapA (rGapA) or Gap (rGap) proteins each time, followed by purification of polyclonal antibodies from serum using the Protein A Resin column (Genscript, China) following the manufacturer’s instructions.

### Gene silencing

The transcription of *gapA* and *gap* was silenced using the CRISPRi system according to the previous methods [12,13]. The CRISPR spacer sequence for dCas9 nucleases was chosen using Cas-Designer (http://www.rgenome.net/cas-designer/), followed by insertion of the spacer sequence into pTargetF by the inverse PCR. The information of the CRISPRi target site sequences is listed in Table 2. The linearized recombinant pTargetF was ligated by T4 DNA ligase for 16 h, followed by transfection of recombinant pTargetF into *C. sakazakii* ATCC 29544 chemically competent cells transfected with pdCas9. Gene silencing was performed using 1 μM of anhydrotetracycline to catalyse the co-expression of the dCas9/sgRNA complex (gene-knockdown treat, KT), while the control check (CK) strain was cultured without the addition of anhydrotetracycline.

**Table 2.** The CRISPRi target site sequences used in this study.

Gene	CRISPRi target site sequence	GC content (%)	Out-of-frame score	Position in the template strand of gene
*gapA*	GGTCAACAACGGATACGTTC	50.0	71.6	270–289
*gap*	ATTCGTTATCATACCAGGCG	45.0	72.1	60–79

### Assessment of gene silencing efficiency

The effect of gene silencing on mRNA expression was verified by a real-time reverse transcription PCR (RT-PCR) assay according to the previous study [4,19] with slight modifications. Bacterial cells were collected by centrifugation for 5 min at 3341 × g, followed by extraction of total RNA using the SteadyPure RNA extraction kit (Agbio, China). Total RNA was quantified using a Colibri Microvolume Spectrometer (Berthold, Germany), and cDNA was synthesized using the Evo M-MLV reverse transcription kit (Agbio, China). RT-PCR assay was performed using a Mastercycler ep realplex (Eppendorf, Germany) and SYBR Green Pro Taq HS qPCR kit (Agbio, China). The fold change in gene expression was calculated by the ΔΔCt method and normalized to the fold change of the 16S rRNA gene.

The effect of gene silencing on protein expression was assessed by SDS-PAGE and Western blot (WB) assays and performed according to the previous study [20] with slight modifications. Bacterial cells were collected by centrifugation for 5 min at 3341 × g and the cell-broken supernatant was collected by centrifugation for 10 min at 13,362 × g. Proteins in the supernatant were separated using 12% SDS-PAGE gels and then transferred onto a 0.22 μM polyvinylidene fluoride membrane (Millipore, USA) using a TRANS-BLOT System (Bio-Rad, USA). The membrane was blocked with Tris-buffered saline containing 0.1% Tween 20 (TBST) and 5% skimmed milk for 1 h at room temperature, followed by incubation with prepared primary antibodies against GapA and Gap (1:10000 dilution) for 2 h, with anti-rabbit IgG-HRP secondary antibody (1:5000 dilution, Solarbio, China) for 1 h. Protein-antibody complexes were imaged using a Chemiluminescence Image Analysis System (Tanon, China), and protein expression was assessed by calculating the pixel intensities of the bands using ImageJ (National Institutes of Mental Health, USA).

### Assessment of growth curve and viability

The OD_600_ of bacterial cells was recorded at 30 min intervals to construct growth curves. In addition, 100 μL of the bacterial suspension was diluted and dispersed on an LB agar plate, and the colony-forming unit (CFU) was counted to determine the viability of bacterial cells.

### Assessment of morphology and swimming motility

Bacterial cells were imaged using a Scanning Electron Microscope (SEM, Hitachi, Japan). Briefly, bacterial cells with the same viability were fixed with 2.5% glutaraldehyde for 12 h at 4°C and then washed with 30%, 50%, 70%, 80%, 90%, and 100% ethanol for 10 min, respectively, followed by drops on a copper mesh and dried in a filter before imaging.

To assess the swimming motility, special LB agar plates containing 1 g/100 mL peptone, 0.5 g/100 mL yeast extract, 1 g/100 mL NaCl, and 0.3% agar powder (wt/vol) were prepared. 10 μL of bacterial cells was dropped in the centre of the plate, followed by incubation for 12 h at 37°C. Swimming motility was assessed based on the diameter of the swimming zone.

### Cell culture

Human ileocecal adenocarcinoma cell line (HCT-8) and human intestinal epithelial cell line-6 (HIEC-6) cells were cultured in RPMI 1640 medium (GIBCO, USA) supplemented with 10% foetal bovine serum (FBS), 100 U/mL of streptomycin, and 100 U/mL of penicillin G and incubated in a humidified incubator at 37°C with 5% CO_2_.

### Assessment of cytotoxicity

The cytotoxicity of recombinant proteins on both cells was assessed by the Thiazolyl Blue Tetrazolium Bromide (MTT) method described previously [21] with slight modifications. Cells forming confluent monolayers in 96-well plates were continued to be incubated for 24 and 48 h in RPMI 1640 medium (with 2% FBS) supplemented with 2, 4, 8, 16, and 32 μg of the recombinant proteins, followed by 20 μL of MTT solution (5 μg/mL, SolarBio, China) added to each well for another 3 h. The formazan in each well was completely dissolved with DMSO (SolarBio, China) after removing the culture supernatants, and the absorbance was determined at 490 nm using a Varioskan™ LUX Multimode Microplate Reader Comes Equipped (Thermo Fisher Scientific, USA). The fold change in absorbance was calculated to determine the relative survival rate.

### Assessment of adhesion and invasion

The capability of bacteria to adhere to and invade both cells was assessed according to the previous methods [22,23] with slight modifications. 2 × 10^4^ to 6 × 10^4^ cells were inoculated into 24-well plates and cultured to form confluent monolayers. The bacterial cells washed with PBS were resuspended in fresh RPMI 1640 medium and adjusted to an OD_600_ of 0.2 (~1 × 10^7^ CFU). Bacterial cells with the same viability were inoculated into 24-well plates so that each eukaryotic cell was infected by ~10 bacterial cells, and the total volume of each well was supplemented to 1 mL. To assess adhesion, after 1.5 h of co-culture, the cells were washed three times with PBS to remove non-adherent bacteria cells, followed by lysing with 0.1% Triton X-100 to count CFUs (adherent and intracellular bacteria).

To assess invasion, after 1.5 h of co-culture, the bacteria-containing medium was replaced with fresh RPMI 1640 medium supplemented with 100 μg/mL gentamicin. The cells were continued to be cultured for 1.5 h to kill adherent bacteria, followed by washing, lysing, and counting of CFUs (intracellular bacteria).

An additional 50 μg of inactivated or active recombinant proteins were added to each well to assess the effect on *C. sakazakii* adhesion to and invasion of cells. The fold change in CFUs was calculated for the KT strain versus the CK strain to determine the relative adhesion and invasion rates.

### Assessment of NF-κB phosphorylation by WB assay

The expression levels of proteins related to the NF-κB signalling pathway was assessed by WB assay with monoclonal antibodies, including Anti-IκB alpha (ab32518), Anti-IκB alpha (phospho S36) (ab133462), Anti-NF-κB p105/p50 (ab32360), Anti-NF-κB p65 (ab16502) (Abcam, UK), and β-actin (AC004) (ABclonal, China). The proteins from both cells were collected using the Native lysis Buffer (SolarBio, China) followed by SDS-PAGE and WB assays. The fold change in pixel intensity was calculated for the rGapA (or rGap) versus the control and normalized to that of β-actin to determine the relative adhesion and invasion rates.

### Assessment of the inflammatory cytokine secretion by ELISA method

The secretion levels of inflammatory cytokines such as TNFα, IL-6, and IL-8 in culture supernatants were assessed using the ELISA Kit (NovusBio, USA) according to the manufacturer’s instructions.

### Assessment of the expression of inflammatory cytokines and tight junction proteins (TJs) by RT-PCR assay

The expression levels of inflammatory factors and TJs in intestinal cells were assessed by RT-PCR assay. Cells forming confluent monolayers in 24-well plates were incubated for 24 h in RPMI 1640 medium (with 2% FBS) supplemented with 50 μg of inactivated and active recombinant protein, followed by total RNA extraction and assessment of inflammatory cytokines (including interleukin 6 (IL-6), IL-8, IL-1β, and tumour necrosis factor-alpha (TNFα)) and TJs (including occludin (OCLN), claudin-1 (CLDN-1), CLDN-2, zonula occludens-1 (ZO-1), and ZO-2). The fold change in gene expression was calculated by the ΔΔCt method and normalized to the fold change of GAPDH.

### Neonatal rat virulence assay

Neonatal rats, obtained from Spyford Biotechnology Co., Ltd., were selected to construct a systemic infection model of *C. sakazakii*, as described by the previously described method [24] with slight modifications. Twenty-eight 3-day-old neonatal rats were randomly divided into four groups (seven neonatal rats per group, and cages were randomly placed) and did not live with their biological mothers: the negative control group (fed with goat’s milk), the positive control group (fed with goat’s milk containing the CK strain), the *gapA* silencing group (fed with goat’s milk containing the *gapA*-KT strain), and the *gap* silencing group (fed with goat’s milk containing the *gap*-KT strain). Among them, bacterial cells washed with PBS were resuspended in goat’s milk and adjusted to ~1 × 10^8^ CFU/100 μL. Each group of neonatal rats was first acclimated for 2 days and given 100 μL of goat’s milk every 4 h, and then 100 μL of prepared sample was fed through intra-oesophageal gavage and again 24 h later.

All neonatal rats were euthanized 48 h after the first feeding of samples, and blood, brain, liver, spleen, kidney, and colon were immediately collected and divided into portions under aseptic conditions, followed by counting of *C. sakazakii* CFUs in blood and homogenized organs (excluding the colon) using the *C. sakazakii* Chromogenic Medium (Hopebiol, China). Five neonatal rats in each group were randomly selected for counting (*n* = 5).

The expression levels of inflammatory cytokines such as IL-1β, IL-6, IL-8, TNFα, and interferon-γ (IFN-γ) in blood was determined by the ELISA Kit (MMBIO, China). The blood of the four neonatal rats in each group was randomly selected for determination (*n* = 4).

Total RNA from brains and colons frozen in liquid nitrogen was extracted to determine the expression levels of inflammatory cytokines (including IL-6, IL-10, IL-1β, TNFα, and IFN-γ) and TJs (including OCLN, CLDN-1, CLDN-2, ZO-1, and ZO-2). The brains and colons of the three neonatal rats in each group were randomly selected for determination (*n* = 3). The fold change in gene expression was normalized to that of GAPDH.

Brains and colons fixed in 10% formalin solution were embedded with paraffin and stained with haematoxylin-eosin. Brain and colon damage was imaged using an ECLIPSE CI upright microscope and a DS-U3 imaging system (NIKON, Japan). All animal experiments were approved by the Institutional Animal Care and Use Committee of the Tianjin University of Science & Technology (20221112) and performed under the guidance. This study was conducted in compliance with the ARRIVE guidelines to ensure transparent and complete reporting of research involving animals.

### Statistical analysis

The statistical analyses were assessed using GraphPad Prism 8.0.2 (GraphPad Software, USA) and SPSS 27.0 (SPSS Inc., USA) and performed using a one-way analysis of variance (ANOVA) and Student’s t tests as the mean ± standard deviation (SD) of at least three independent experiments. *P*-values of 0.05 or less were considered statistically significant and were expressed as *p < 0.05, **p < 0.01, ***p < 0.001, and ****p < 0.0001, and ns for not significant.

## Results

### GapA and Gap are conserved proteins in different organisms

GapA is widely regarded as a conserved protein, the conservation between the amino acid sequences of both GapA and Gap proteins of *C. sakazakii* and these two proteins from different organisms was assessed (Figure S1). The GapA sequences are highly conserved in many of the organisms listed, and the GapA sequences of the seven *Cronobacter* spp. were particularly highly conserved, with up to 100% identity (Figure S1a). In addition, the GapA sequences of several different genera in bacteria are also highly conserved, up to 97.89%. The Gap sequences are also conserved in these organisms, especially in *Cronobacter* spp. with up to 99.70% identity (Figure S1b). Compared to the GapA, Gap is less conserved than GapA.

### Gene silencing efficiently inhibit gene transcription and protein expression

**Figure 1.**
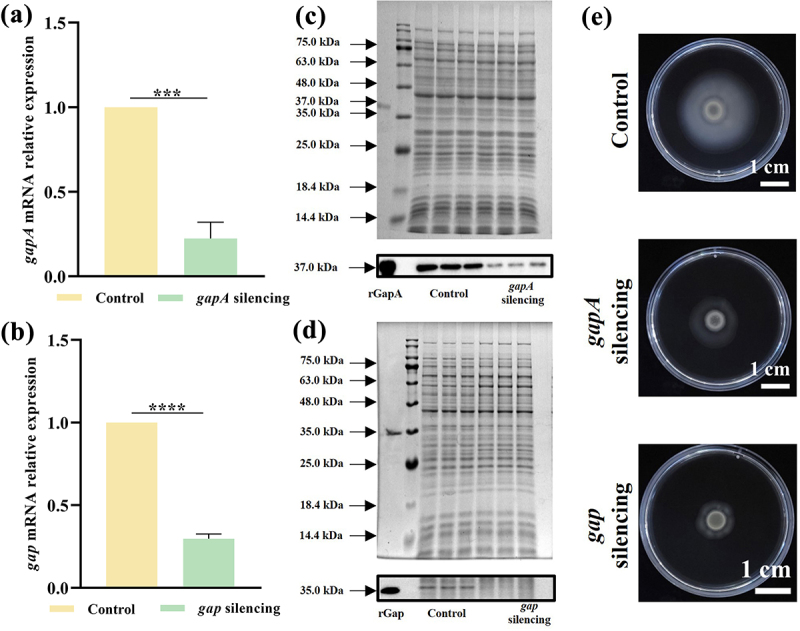
Gene silencing reduces the expression of GapA and Gap and reduces the swimming motility of *C. sakazakii*.

Since GapA is indispensable in numerous organisms such as *C. sakazakii*, the virulence properties of GapA and Gap were investigated by gene silencing, and the silencing efficiency was verified by RT-PCR and WB assays. Gene silencing efficiently inhibited gene transcription, with a 77.68% and 70.37% reduction in the expression levels of *gapA* and *gap*, respectively (Figure 1a,b). These two recombinant proteins prepared with molecular weights of ~37.0 and ~35.0 kDa were recognized by the prepared polyclonal antibodies against rGapA and rGap, respectively, and the WB assay showed that the expression levels of GapA and Gap were reduced by 70.73% and 40.10%, respectively, after gene silencing (Figure 1c,d).

(a and b) RT-PCR assay of the expression of *gapA* and *gap*. The fold change in gene expression was calculated by the ΔΔCt method and normalized to the fold change of the 16S rRNA gene. The results shown indicate the mean ± SD of three independent experiments. ***P < 0.001 vs. control. (c) SDS-PAGE and WB assays of cell-broken supernatants of CK strain (control) and *gapA*-KT strain (*gapA* silencing) to verify the expression-level change of GapA, and the antibody used was the prepared polyclonal antibody anti-rGapA. (d) SDS-PAGE and WB assays of cell-broken supernatants of CK strain and *gap*-KT strain (*gap* silencing) to verify the expression-level change of Gap, and the antibody used was the prepared polyclonal antibody anti-rGap. (e) The diameter of the swimming zone was used to assess swimming motility. The scale bars represent 1 cm.

### GapA and Gap affect bacterial vitality

Silencing with normal transcription of genes may affect bacterial growth, including growth rate and viability. The time required for *C. sakazakii* to grow to the logarithmic phase increased following a decrease in the expression levels of GapA and Gap (Figure S2a). The CK strain grew to an OD_600_ of 0.6 after ~2.5 h of culture compared with ~3.5 h and ~4.0 h for the *gapA*-KT and *gap*-KT strains, respectively. Bacterial cell volume was adjusted based on the calculated viable bacteria to ensure that the CK and KT strains with the same viability were used in the experiments. The CFUs of the *gapA*-KT strain were significantly less than that of the CK strain at OD_600_ at 0.4, while the CFUs of the *gap*-KT strain were even significantly less than that of the *gapA*-KT strain at OD_600_ of 0.4, 0.6, 0.8, 1.0, and 1.2 (Figure S2b).

### GapA and Gap affect bacterial morphology and swimming motility

Gene silencing also affected bacterial morphology. Some *gapA*-KT strains had shorter cell lengths compared with the CK strains, and conversely, some *gap*-KT strains grew longer and even wrinkled (Figure S2c). These results indicate that the normal expression of both moonlighting proteins is indispensable for maintaining the normal growth of bacterial cells.

Swimming motility is one of the main characteristics of *C. sakazakii*, and the diameter of the swimming zone of the *gapA*-KT and *gap*-KT strains was reduced by 48.05% and 61.76%, respectively, compared to the CK strain (Figure 1e). Thus, silenced expression of both proteins reduced the swimming motility of *C. sakazakii*.

### GapA and Gap do not exhibit severe cytotoxicity

The cytotoxicity of the two recombinant proteins was determined prior to the assessment of adhesion and invasion. Both recombinant proteins were co-cultured with cells for 24 and 48 h to determine cytotoxicity (Figure S3a-d). The addition of 32 μg of recombinant protein did not result in more than 50% cell death after 24 and 48 h of co-culture, and therefore neither recombinant protein has severe cytotoxicity to either cell.

### GapA and Gap enhance *C. sakazakii* adhesion to and invasion of intestinal cells

**Figure 2.**
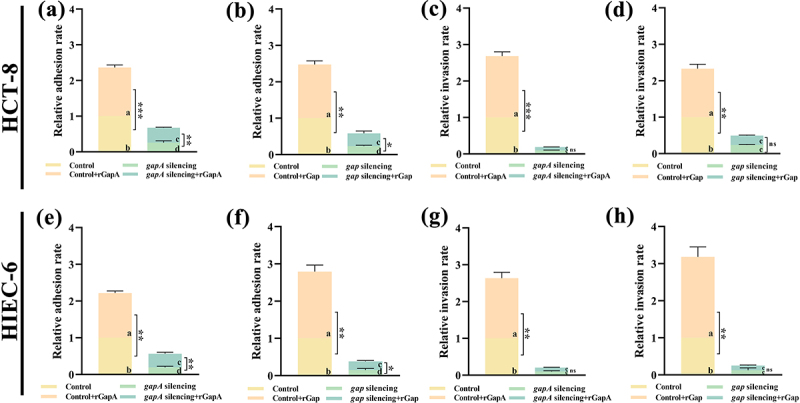
GapA and Gap enhance *C. sakazakii* adhesion to and invasion of intestinal cells.

*C. sakazakii* is able to colonize intestinal cells, so the virulence properties of GapA and Gap on bacterial adhesion to and invasion of both cells were investigated (Figure 2a-h). The relative adhesion and invasion rates of the *gapA*-KT and *gap*-KT strains were reduced by more than 74.21% compared with the CK strain, and the additional active recombinant proteins enhanced the adhesion capability of these two strains, but not the invasion. Surprisingly, both active recombinant proteins were able to enhance the CK strain adhesion to and invasion of both cells. These results indicate that these two proteins play key roles in enhancing *C. sakazakii* adhesion to and invasion of intestinal cells.

In HCT-8 cell adhesion and invasion, relative adhesion rates of (a) CK (control) and *gapA*-KT (*gapA* silencing) strains and (b) CK and *gap*-KT (*gap* silencing) strains with or without protein addition; relative invasion rates of (c) CK and *gapA*-KT strains and (d) CK and *gap*-KT strains with or without protein addition. In HIEC-6 cell adhesion and invasion, relative adhesion rates of (e) CK and *gapA*-KT strains and (f) CK and *gap*-KT strains with or without protein addition; relative invasion rates of (g) CK and *gapA*-KT strains and (h) CK and *gap*-KT strains with or without protein addition. The fold change of CFU was calculated for the KT strain against the CK strain to determine the relative adhesion/invasion rate. The results shown indicate the mean ± SD of three independent experiments. *P < 0.05; **P < 0.01; ***P < 0.001; ns indicates not significant. ^a,b,c,d^ Statistical analysis of ANOVA and different letters indicate *P* < 0.05: the maximum fold change in each figure was defined as “a”; the closest fold change significantly different from “a” was defined as “b”, and not significantly different was also defined as “a”; the closest fold change significantly different from “a” and “b” was defined as “c”, otherwise it was also defined as “b”; the closest fold change significantly different from “a”, “b”, and “c” was defined as “d”, otherwise it was also defined as “c”.

### GapA and Gap enhance NF-κB phosphorylation

**Figure 3.**
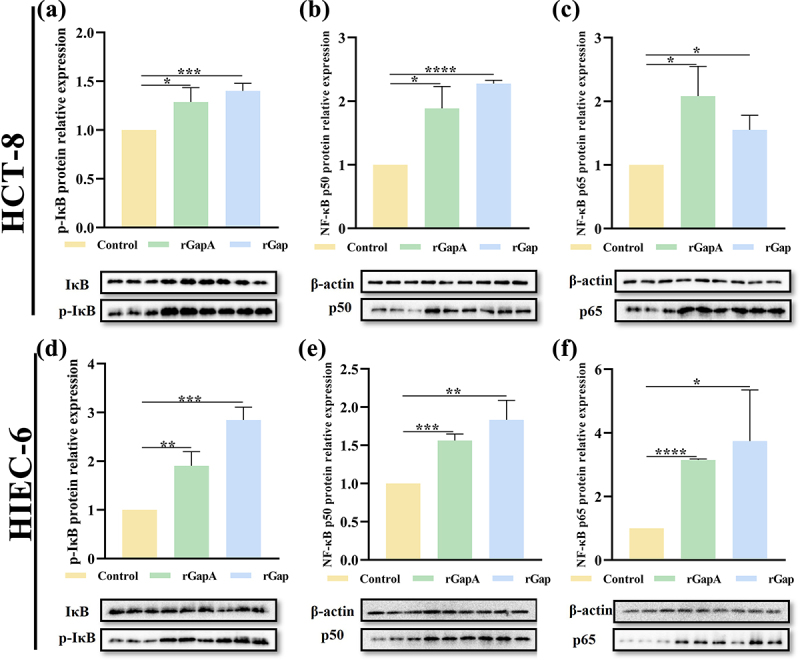
GapA and Gap induce phosphorylation of NF-κB signalling pathway in intestinal cells.

*C. sakazakii* can enhance the phosphorylation of NF-κB in intestinal cells and promote the release of inflammatory cytokines, which contribute to host cell colonization. Therefore, it was assessed whether GapA and Gap have the property of enhancing NF-κB phosphorylation. rGapA and rGap induced increased expression level of p-IκB in HCT-8 cells and at a higher level in HIEC-6 cells (Figure 3a and d). rGapA and rGap induced increased expression levels of NF-κB p50 subunit and p65 subunit in both cells (Figure 3b, c, and e), especially inducing an increase in the expression levels of p65 subunit in HIEC-6 cells by 215.17% and 273.94%, respectively (Figure 3f). The results indicate that the two recombinant proteins play similar roles in eliciting and enhancing phosphorylation and nuclear translocation of the NF-κB signalling pathway.

rGapA and rGap significantly induced increased expression levels of (a) p-IκB, (b) p50 subunit, and (c) p65 subunit in NF-κB signalling pathway in HCT-8 cells. rGapA and rGap significantly induced increased expression levels of (d) p-IκB, (e) p50 subunit, and (f) p65 subunit in NF-κB signalling pathway in HIEC-6 cells. The fold change in pixel intensities was normalized to that of β-actin. The results shown indicate the mean ± SD of three independent experiments. *P < 0.05 vs. control; **P < 0.01 vs. control; ***P < 0.001 vs. control; ****P < 0.0001 vs. control.

### GapA and Gap induce the secretion of inflammatory cytokines

**Figure 4.**
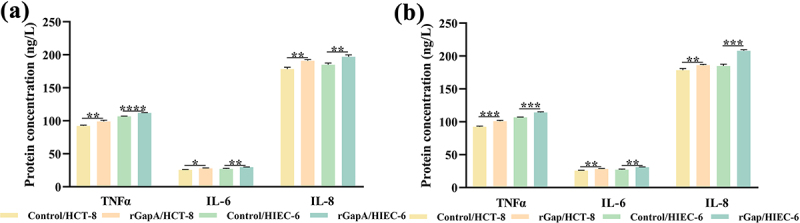
GapA and Gap induce the secretion of TNFα, IL-6, and IL-8 in cell culture supernatants.

The secretion levels of inflammatory cytokines induced by rGapA and rGap were further assessed by ELISA method. Both rGapA and rGap induced an increase in the secretion levels of TNFα, IL-6, and IL-8 in both cells, and in particular, the rGap induced a 14.05% and 12.52% increase in the secretion levels of IL-6 and IL-8, respectively, in HIEC-6 cells (Figure 4a and b).

(a) rGapA induces increased secretion levels of TNFα, IL-6, and IL-8 in cell culture supernatants of HCT-8 and HIEC-6 cells. (b) rGap induces increased secretion levels of TNFα, IL-6, and IL-8 in cell culture supernatants of HCT-8 and HIEC-6 cells. The results shown indicate the mean ± SD of three independent experiments. *P < 0.05 vs. control; **P < 0.01 vs. control; ***P < 0.001 vs. control; ****P < 0.0001 vs. control.

### GapA and Gap induce the expression of inflammatory cytokines and TJs

In addition, the expression-level changes of four inflammatory cytokines (i.e. IL-6, IL-8, IL-1β, and TNFα) in both cells were determined by RT-PCR assay. rGapA was able to induce increased expression levels of IL-1β and TNFα in HCT-8 cells and IL-8 and TNFα in HIEC-6 cells, respectively, and rGap was able to induce increased expression levels of IL-8, IL-1β, and TNFα in HCT-8 cells and IL-6 in HIEC-6 cells, respectively (Figure S4a and c). Both rGapA and rGap induced a decrease in the expression levels of OCLN and ZO-2 in both cells, and they also led to a significant decrease in the expression level of CLDN-1 in HIEC-6 cells (Figure S4b and d). These results indicate that the two recombinant proteins can induce increased expression levels of several inflammatory cytokines (especially TNFα) in intestinal cells and disrupt the intestinal epithelial barrier integrity.

### GapA and Gap are vital for the full pathogenicity of *C. sakazakii* in neonatal rats

**Figure 5.**
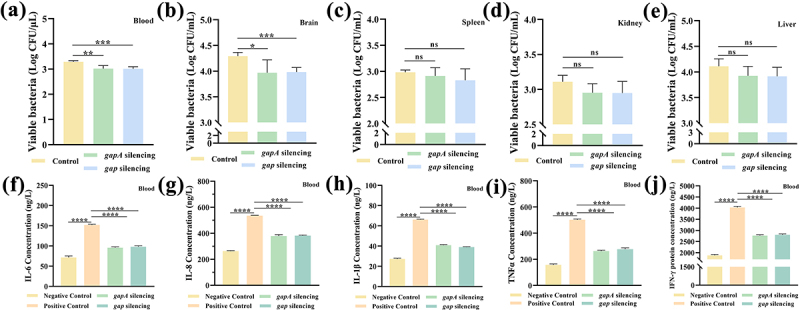
GapA and Gap enhance bacterial infection in neonatal rats and induce inflammatory cytokine expression.

*C. sakazakii* is able to colonize the intestinal barrier and cause systemic infection. The invasion capability of the CK and KT strains was assessed by counting the CFUs of *C. sakazakii* in blood and homogenate tissues, including the brain, spleen, kidney, and liver. Compared with the CK strain, the CFUs of the *gapA*-KT and *gap*-KT strains that invaded the blood and brain were significantly reduced, but there was no significant change in the CFUs of bacteria that invaded the spleen, kidney, and liver (Figure 5a-e).

CFUs of CK (control), *gapA*-KT (*gapA* silencing), and *gap*-KT (*gap* silencing) strains invading the (a) blood, (b) brain, (c) spleen, (d) kidney, and (e) liver of neonatal rats. Production of (f) IL-6, (g) IL-8, (h) IL-1β, (i) TNFα, and (j) IFN-γ in the blood. Negative control (fed with goat’s milk); Positive control (fed with goat’s milk containing CK strain); *gapA* silencing group (fed with goat’s milk containing *gapA*-KT strain); *gap* silencing group (fed with goat’s milk containing *gap*-KT strain). The results shown indicate the mean ± SD of four independent experiments. (a-e) *P < 0.05 vs. control; **P < 0.01 vs. control; ***P < 0.001 vs. control; ns indicates not significant. (f-j) ****P < 0.0001 vs. positive control.

*C. sakazakii* infection resulted in a significant increase in the secretion levels of five inflammatory cytokines in the blood of neonatal rats, but the silenced expression of both proteins reduced the induction capability (Figure 5f-j). Importantly, the expression levels of TNFα were also highest in the CK, *gapA* silencing, and *gap* silencing groups compared to the negative control group.

The expression levels of inflammatory cytokines and TJs in the brain and colon were also determined. Both silenced expression of GapA and Gap decreased the expression levels of IL-6 and IL-1β induced by *C. sakazakii* in the brain and colon (Figure S5a and c). Silenced expression of GapA weakened the capability of the *gapA*-KT strain to decrease the expression levels of ZO-2 in the brain and CLDN-2 in the colon, respectively, and silenced expression of Gap weakened the capability of the *gap*-KT strain to decrease the expression levels of CLDN-1 and ZO-1 in the brain and CLDN-1 and CLDN-2 in the colon, respectively (Figure S5b and d).

**Figure 6.**
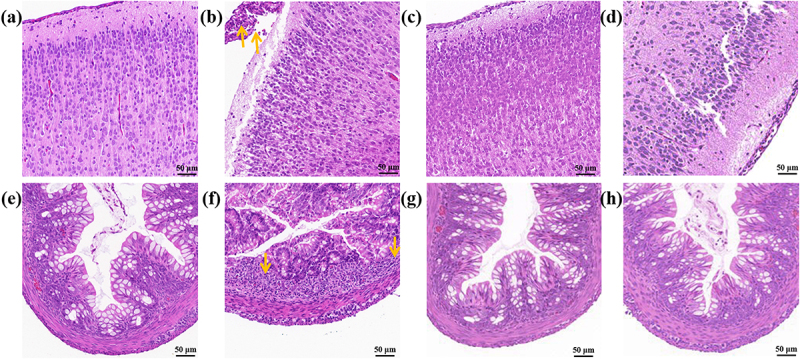
GapA and Gap are involved in the pathogenicity of *C. sakazakii*.

Finally, brain and colon damage caused by the CK, *gapA*-KT, and *gap*-KT strains were evaluated. When gavaged goat’s milk without *C. sakazakii*, the brain tissue and leptomeninges of the neonatal rats were intact, no inflammatory cell infiltrating was observed, and no necrosis or neuronal degeneration was observed in the cerebral cortex (Figure 6a). Mild symptoms of meningitis and inflammatory cells predominantly lymphocytes were observed in the leptomeninges when gavaged goat’s milk containing the CK strain, as indicated by the orange arrows (Figure 6b). In contrast, neither *gapA*-KT nor *gap*-KT strains caused meningitis symptoms and inflammatory cell infiltrating in the brain (Figure 6c and d). No structural changes or inflammatory cell infiltrating were observed in the colon when gavaged goat’s milk (Figure 6e). In addition, the epithelial cells and crypts in the mucosal layer were neatly arranged and tightly packed, and there was no sign of oedema below the mucosal layer. The CK strain caused moderate damage to the colon structure of neonatal rats, with large numbers of inflammatory cells, predominantly neutrophils, accumulating in the colon, as indicated by the orange arrows (Figure 6f). Neither *gapA*-KT nor *gap*-KT strains caused serious damage to the colon (Figure 6g and h). The results indicated that both proteins also play indispensable roles in enhancing the capability of *C. sakazakii* to invade neonatal rats and in stimulating host inflammatory responses.

Brains of neonatal rats that were gavaged with goat’s milk containing (a) nothing, (b) CK strain, (c) *gapA*-KT strain, and (d) *gap*-KT strain. Colons of neonatal rats that gavage with goat’s milk containing (e) nothing, (f) CK strain, (g) *gapA*-KT strain, and (h) *gap*-KT strain. The scale bars represent 50 μm. Orange arrows point to (b) inflammatory cells predominantly lymphocytes in brains and (f) inflammatory cells predominantly neutrophils in colons, respectively.

## Discussion

Glycolysis and gluconeogenesis are basic and indispensable metabolic processes that promote cellular homoeostasis [25]. To take advantage of the finite-size bacterial genome to enhance bacterial pathogenicity, key moonlighting enzymes in the glycolysis pathway such as GapA and gluconeogenesis pathway such as Gap can perform a variety of unrelated functions depending on their localization in bacterial cells, and their potential virulence properties are of widespread interest [26–28]. GapA exhibits virulence properties when it is located on the bacterial cell surface or secreted extracellularly, which has been demonstrated in some bacterial pathogens [29]. For example, extracellular GapA of *Mycobacterium tuberculosis* can bind to and activate cell surface receptors (e.g. plasminogen), further facilitating pathogen-intestinal epithelial cell interactions and pathogen transport [5]. Our data demonstrated that silenced expression of GapA and Gap reduced the capability of *C. sakazakii* to adhesion to and invasion of intestinal cells, and these two recombinant proteins can enhance *C. sakazakii* adhesion to intestinal cells. We speculate that GapA and Gap of *C. sakazakii* may also have similar virulence properties that bind to and activate host cell surface receptors. GapA and Gap of *C. sakazakii* may increase the connection between *C. sakazakii* and host intestinal cells by activating more surface receptors, thus leading to enhanced adhesion capability. On the other hand, GapA and Gap activated stronger immune responses in intestinal cells and disrupted the tight junctions of intestinal barriers, which may also result from the binding of the recombinant proteins to surface receptors, which further contributed to the increased invasion capability of *C. sakazakii*.

After the construction of the dCas9/sgRNA complex strains, silencing the normal expression of either of these two genes disrupted bacterial cell homoeostasis and resulted in a reduced growth rate of *C. sakazakii*. It is worth noting that the reduction in *gap* expression had more severe effects on bacterial growth rate and morphology than *gapA*. Since the Gap functions inversely from the NADP-dependent GapA, it also differs from D-erythrose-4-phosphate dehydrogenase Epd in the gluconeogenesis pathway (amino acid similarity ~41.89%). We speculate that Gap has the potential to partially restore glycolysis impaired by disruption of GapA expression. In contrast, due to the specificity of Gap, the efficiency of GapA or Epd in repairing impaired glycolysis or gluconeogenesis pathways is extremely low. Silencing the normal expression of either of these two proteins also resulted in a significant reduction in the swimming motility of *C. sakazakii*, which may also be the result of a disruption of bacterial cellular homoeostasis. Bacteria such as *C. sakazakii* require energy, such as ATP, to control the rotation of the flagellum [30]. The moonlighting enzymes GapA and Gap are key metabolic enzymes in the glycolysis and gluconeogenesis pathways, respectively, and both directly affect the production of ATP in bacterial cells. Although there is no direct evidence, we speculate that GapA and Gap can also interact with some flagellum-related proteins, affecting the formation or motility of flagellum, and thus bacterial swimming motility and virulence. This highlights the importance of GapA and Gap in maintaining bacterial cellular homoeostasis and full virulence.

In addition, the results of the animal experiment of this study also demonstrated the capability of *C. sakazakii* to penetrate the intestinal barrier and establish infection in multiple organs. The results showed that the systemic infection capability of the *gapA*-KT and *gap*-KT strains was weakened with the silenced expression of these two proteins. The silenced expression of GapA or Gap may, on the one hand, directly led to a reduction in the capability of *C. sakazakii* to colonize the intestinal cells and stimulate host inflammatory responses, thereby further reducing the pathogenicity. On the other hand, it may also be that since they are moonlighting enzymes, the silenced expression of them affected the full function of virulence factors such as OmpA, OmpX, and FliC during the pathogenic process of *C. sakazakii* [31].

As part of the innate immune response, toll-like receptors (TLRs) recognize pathogens and relay signals to the adapter protein MyD88, which then activates TAK1, thereby promoting the degradation of NF-κB kinase inhibitor IKK. This results in phosphorylation of IκBα, p50 subunit, and p65 subunit, resulting in the release of inflammatory cytokines [32]. *C. sakazakii* has been reported to have the potential to cause NEC by stimulating TLRs as well as to stimulate the NF-κB signal pathway to regulate inflammatory responses [33,34]. The results showed that these two recombinant proteins promoted the activation of the NF-κB signalling pathway by enhancing IκB phosphorylation and releasing the p50 subunit and p65 subunit of the NF-κB signalling pathway. Several studies have demonstrated that increased expression and secretion levels of various inflammatory cytokines such as TNFα, IL-6, IL-8, and IL-1β are associated with the activation of the NF-κB pathway [35]. The results of this study showed that the stimulation of these two recombinant proteins induced increased secretion levels of TNFα, IL-6, and IL-8. Notably, IL-6 has pro-inflammatory and anti-inflammatory properties and is involved in most of the immune responses, and its anti-inflammatory property contributes significantly to the maintenance of intestinal homoeostasis [36]. Thus, it is likely that the increased secretion level of IL-6 was mediated by the inhibitory effect of IL-6 as an anti-inflammatory cytokine on TNF-α and other inflammatory cytokines. Although IL-6 has anti-inflammatory properties, it can also be commonly used to evaluate changes in host inflammation [37].

In conclusion, this study demonstrates that the silenced expression of the moonlighting enzymes GapA and Gap reduces the capabilities of *C. sakazakii* to adhere to and invade intestinal cells and reduces its pathogenicity in neonatal rats. In addition, the recombinant GapA and Gap exhibit important virulence properties that enhance *C. sakazakii* adhesion and induce inflammatory responses in intestinal cells.

## Highlights

- GapA and Gap contributed to *C. sakazakii* adhesion to and invasion of intestinal cells.
- GapA and Gap induced NF-κB signalling pathway phosphorylation and inflammatory responses in intestinal cells.
- GapA and Gap enhanced the capability of *C. sakazakii* to infect the blood and brain of neonatal rats.

## Supplementary Material

Figure S2.jpg

Figure S1.jpg

Figure S3.jpg

Figure S5.jpg

Author_Checklist_Full.pdf

Figure S4.jpg

## Data Availability

The data that support the findings of this study are openly available in https://doi.org/10.6084/m9.figshare.25907515.v3.

## References

[1] Drolia R, Bryant DB, Tenguria S, et al. *Listeria* adhesion protein orchestrates caveolae-mediated apical junctional remodeling of epithelial barrier for *listeria monocytogenes* translocation. MBio. 2024;15(3):e0282123. doi: 10.1128/mbio.02821-23 38376160 PMC10936185

[2] Veetilvalappil VV, Manuel A, Aranjani JM, et al. Pathogenic arsenal of *Pseudomonas aeruginosa*: an update on virulence factors. Future Microbiol. 2022;17(6):465–14. doi: 10.2217/fmb-2021-0158 35289684

[3] Lipke PN, Ragonis-Bachar P. Sticking to the subject: multifunctionality in microbial adhesins. JoF. 2023;9(4):419. doi: 10.3390/jof9040419 37108873 PMC10144551

[4] Gani Z, Boradia VM, Kumar A, et al. *Mycobacterium tuberculosis* glyceraldehyde-3-phosphate dehydrogenase plays a dual role—As an adhesin and as a receptor for plasmin(ogen). Cell Microbiol. 2021;23(5):e13311. doi: 10.1111/cmi.13311 33486886

[5] Wang J, Li Y, Pan L, et al. Glyceraldehyde-3-phosphate dehydrogenase (GAPDH) moonlights as an adhesin in *mycoplasma hyorhinis* adhesion to epithelial cells as well as a plasminogen receptor mediating extracellular matrix degradation. Vet Res. 2021;52(1):80. doi: 10.1186/s13567-021-00952-8 34082810 PMC8173509

[6] Bednarek A, Satala D, Zawrotniak M, et al. Glyceraldehyde 3-phosphate dehydrogenase on the surface of Candida albicans and Nakaseomyces glabratus cells—A moonlighting protein that binds human vitronectin and plasminogen and can adsorb to pathogenic fungal cells via Major adhesins Als3 and Epa6. IJMS. 2024;25(2):1013. doi: 10.3390/ijms25021013 38256088 PMC10815899

[7] Fillinger S, Boschi-Muller S, Azza S, et al. Two glyceraldehyde-3-phosphate dehydrogenases with opposite physiological roles in a nonphotosynthetic bacterium. J Biol Chem. 2000;275(19):14031–14037. doi: 10.1074/jbc.275.19.14031 10799476

[8] Ling N, Li C, Zhang J, et al. Prevalence and molecular and antimicrobial characteristics of *cronobacter* spp. isolated from raw vegetables in China. Front Microbiol. 2018;9:1149. doi: 10.3389/fmicb.2018.01149 29922254 PMC5996200

[9] Jang H, Gopinath GR, Eshwar A, et al. The secretion of toxins and other exoproteins of *Cronobacter*: role in virulence, adaption, and persistence. Microorganisms. 2020;8(2):229. doi: 10.3390/microorganisms8020229 32046365 PMC7074816

[10] Boschi-Muller S, Azza S, Pollastro D, et al. Comparative enzymatic properties of GapB-encoded erythrose-4-phosphate dehydrogenase of escherichia coliand phosphorylating glyceraldehyde-3-phosphate dehydrogenase. J Biol Chem. 1997;272(24):15106–15112. doi: 10.1074/jbc.272.24.15106 9182530

[11] Wang J, Du XJ, Lu XN, et al. Immunoproteomic identification of immunogenic proteins in *cronobacter sakazakii* strain BAA-894. Appl Microbiol Biotechnol. 2013;97(5):2077–2091. doi: 10.1007/s00253-013-4720-5 23371297

[12] Qi LS, Larson MH, Gilbert LA, et al. Repurposing CRISPR as an RNA-guided platform for sequence-specific control of gene expression. Cell. 2013;152(5):1173–1183. doi: 10.1016/j.cell.2013.02.022 23452860 PMC3664290

[13] Larson MH, Gilbert LA, Wang X, et al. CRISPR interference (CRISPRi) for sequence-specific control of gene expression. Nat Protoc. 2013;8(11):2180–2196. doi: 10.1038/nprot.2013.132 24136345 PMC3922765

[14] Feng J, Pan M, Zhuang Y, et al. Genetic epidemiology and plasmid-mediated transmission of *mcr-1* by *Escherichia coli* ST155 from wastewater of long-term care facilities. Microbiol Spectr. 2024;12(3):e03707–23. doi: 10.1128/spectrum.03707-23 38353552 PMC10913736

[15] Letunic I, Bork P. Interactive tree of life (iTOL) v5: an online tool for phylogenetic tree display and annotation. Nucleic Acids Res. 2021;49(W1):W293–W296. doi: 10.1093/nar/gkab301 33885785 PMC8265157

[16] Madureira P, Baptista M, Vieira M, et al. *Streptococcus agalactiae* GAPDH is a virulence-associated immunomodulatory protein. The J Immunol. 2007;178(3):1379–1387. doi: 10.4049/jimmunol.178.3.1379 17237385

[17] Gründel A, Pfeiffer M, Jacobs E, et al. Network of surface-displayed glycolytic enzymes in *Mycoplasma pneumoniae* and their interactions with human plasminogen. Infect Immun. 2016;84(3):666–676. doi: 10.1128/iai.01071-15 PMC4771348 26667841

[18] Talib NAA, Salam F, Sulaiman Y. Development of polyclonal antibody against clenbuterol for immunoassay application. Molecules. 2018;23(4):789. doi: 10.3390/molecules23040789 29596322 PMC6017646

[19] Prieto AI, Hernández SB, Cota I, et al. Roles of the outer membrane protein AsmA of *Salmonella enterica* in the control of *marRAB* expression and invasion of epithelial cells. J Bacteriol. 2009;191(11):3615–3622. doi: 10.1128/jb.01592-08 19346309 PMC2681915

[20] Ferreira E, Giménez R, Aguilera L, et al. Protein interaction studies point to new functions for *Escherichia coli* glyceraldehyde-3-phosphate dehydrogenase. Res Microbiol. 2013;164(2):145–154. doi: 10.1016/j.resmic.2012.11.002 23195894

[21] Han H, You Y, Cha S, et al. Multi-species probiotic strain mixture enhances intestinal barrier function by regulating inflammation and tight junctions in *Lipopolysaccharides* stimulated caco-2 cells. Microorganisms. 2023;11(3):656. doi: 10.3390/microorganisms11030656 36985228 PMC10056128

[22] Lambert MA, Smith SG. The PagN protein of *Salmonella enterica* serovar *Typhimurium* is an adhesin and invasin. BMC Microbiol. 2008;8(1):142. doi: 10.1186/1471-2180-8-142 18778463 PMC2553418

[23] Péchiné S, Denève-Larrazet C, Collignon A. *Clostridium difficile* adhesins. Methods In Mol Biol. 2016;1476:91–101. doi: 10.1007/978-1-4939-6361-4_7 27507335

[24] He X, Zeng Q, Puthiyakunnon S, et al. *Lactobacillus rhamnosus* GG supernatant enhance neonatal resistance to systemic *Escherichia coli* K1 infection by accelerating development of intestinal defense. Sci Rep. 2017;7(1):43305. doi: 10.1038/srep43305 28262688 PMC5338013

[25] Bian X, Jiang H, Meng Y, et al. Regulation of gene expression by glycolytic and gluconeogenic enzymes. Trends Cell Biol. 2022;32(9):786–799. doi: 10.1016/j.tcb.2022.02.003 35300892

[26] Gao J, Tian M, Bao Y, et al. Pyruvate kinase is necessary for *Brucella abortus* full virulence in BALB/c mouse. Vet Res. 2016;47(1):87. doi: 10.1186/s13567-016-0372-7 27561260 PMC5000513

[27] Sullivan MJ, Goh KGK, Thapa R, et al. *Streptococcus agalactiae* glyceraldehyde-3-phosphate dehydrogenase (GAPDH) elicits multiple cytokines from human cells and has a minor effect on bacterial persistence in the murine female reproductive tract. Virulence. 2021;12(1):3015–3027. doi: 10.1080/21505594.2021.1989252 34643172 PMC8667900

[28] Merlo LMF, Peng W, DuHadaway JB, et al. The immunomodulatory enzyme IDO2 mediates autoimmune arthritis through a nonenzymatic mechanism. The J Immunol. 2022;208(3):571–581. doi: 10.4049/jimmunol.2100705 34965962 PMC8770583

[29] Kopeckova M, Pavkova I, Stulik J. Diverse localization and protein binding abilities of glyceraldehyde-3-phosphate dehydrogenase in pathogenic bacteria: the key to its multifunctionality? Front Cell Infect Microbiol. 2020;10:89. doi: 10.3389/fcimb.2020.00089 32195198 PMC7062713

[30] Minamino T, Morimoto YV, Kinoshita M, et al. Membrane voltage-dependent activation mechanism of the bacterial flagellar protein export apparatus. Proc Natl Acad Sci USA. 2021;118(22):e2026587118. doi: 10.1073/pnas.2026587118 34035173 PMC8179193

[31] Shi M, Hou J, Liang W, et al. GAPDH facilitates homologous recombination repair by stabilizing RAD51 in an HDAC1-dependent manner. EMBO Rep. 2023;24(8):e56437. doi: 10.15252/embr.202256437 37306047 PMC10398663

[32] Feng R, Niu Z, Zhang X, et al. *Cryptosporidium parvum* downregulates miR-181d in HCT-8 cells via the p50-dependent TLRs/NF-κB pathway. Vet Parasitol. 2022;305:109710. doi: 10.1016/j.vetpar.2022.109710 35462275

[33] Chen Z, Zhang Y, Lin R, et al. *Cronobacter sakazakii* induces necrotizing enterocolitis by regulating NLRP3 inflammasome expression via TLR4. J Med Microbiol. 2020;69(5):748–758. doi: 10.1099/jmm.0.001181 32209170

[34] Vinay P, Karen C, Balamurugan K, et al. *Cronobacter sakazakii* infection in early postnatal rats impaired contextual-associated learning: a putative role of C5a-mediated nf-κβ and ASK1 pathways. J Mol Neurosci. 2021;71(1):28–41. doi: 10.1007/s12031-020-01622-8 32567007

[35] Zheng J, Ahmad AA, Yang Y, et al. *Lactobacillus rhamnosus* CY12 enhances intestinal barrier function by regulating tight junction protein expression, oxidative stress, and inflammation response in lipopolysaccharide-induced caco-2 cells. IJMS. 2022;23(19):11162. doi: 10.3390/ijms231911162 36232464 PMC9569798

[36] Scheller J, Chalaris A, Schmidt-Arras D, et al. The pro- and anti-inflammatory properties of the cytokine interleukin-6. Biochim et Biophys Acta (BBA) - Mol Cell Res. 2011;1813(5):878–888. doi: 10.1016/j.bbamcr.2011.01.034 21296109

[37] Li Y, Li J, Wang X, et al. Role of intestinal extracellular matrix-related signaling in porcine epidemic diarrhea virus infection. Virulence. 2021;12(1):2352–2365. doi: 10.1080/21505594.2021.1972202 34515624 PMC8451458

