# Supplementary figures and images for: Roles and mechanisms of gapA and gap-encoded proteins in cronobacter sakazakii adhesion to and invasion of intestinal cells and neonatal rats
Source: Virulence. 2024 Dec 30;16(1):2446713. doi: 10.1080/21505594.2024.2446713 (PMC11702931; doi:10.1080/21505594.2024.2446713)

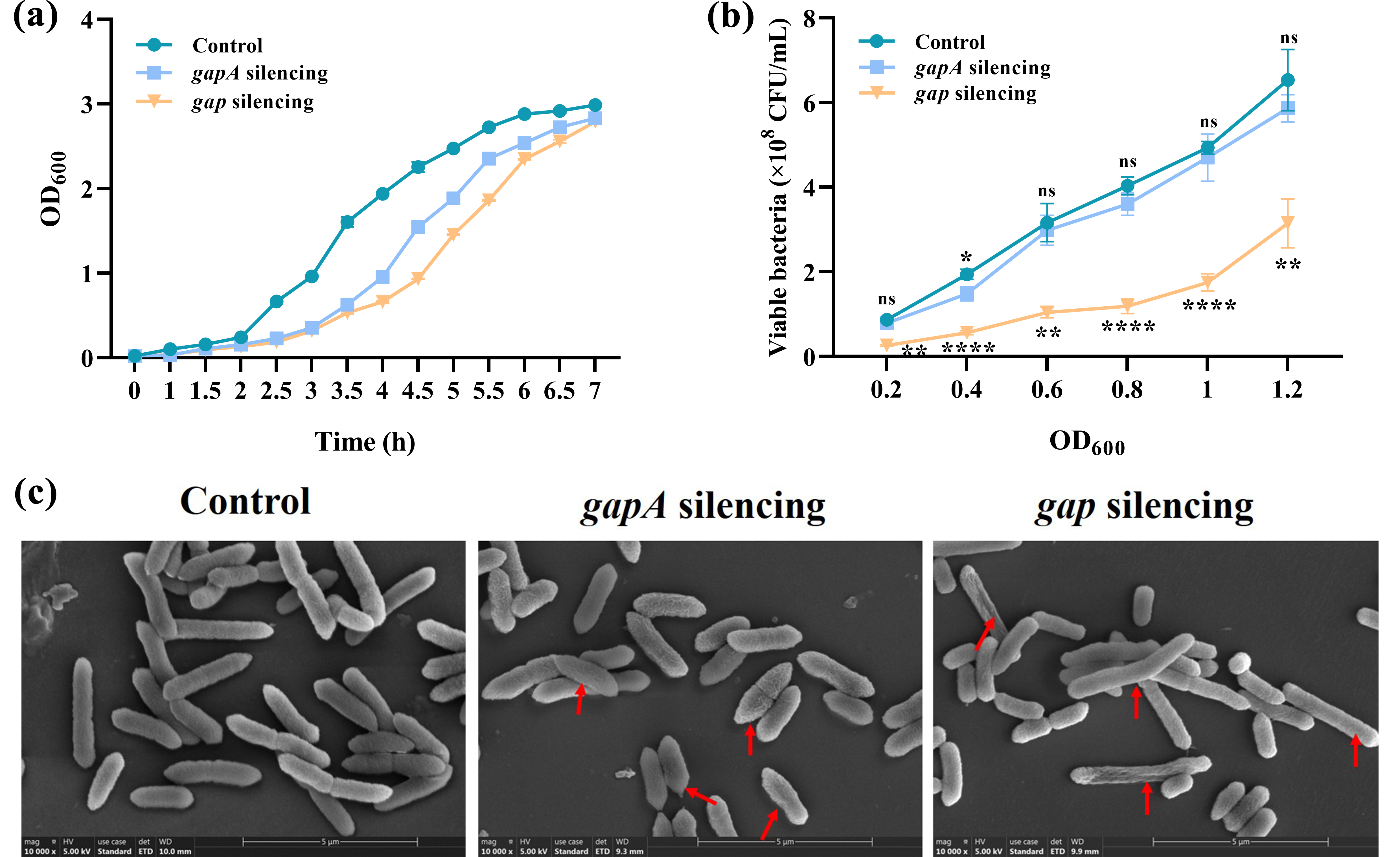

Supplement: Figure S2.jpg [file KVIR_A_2446713_SM3658.jpg]

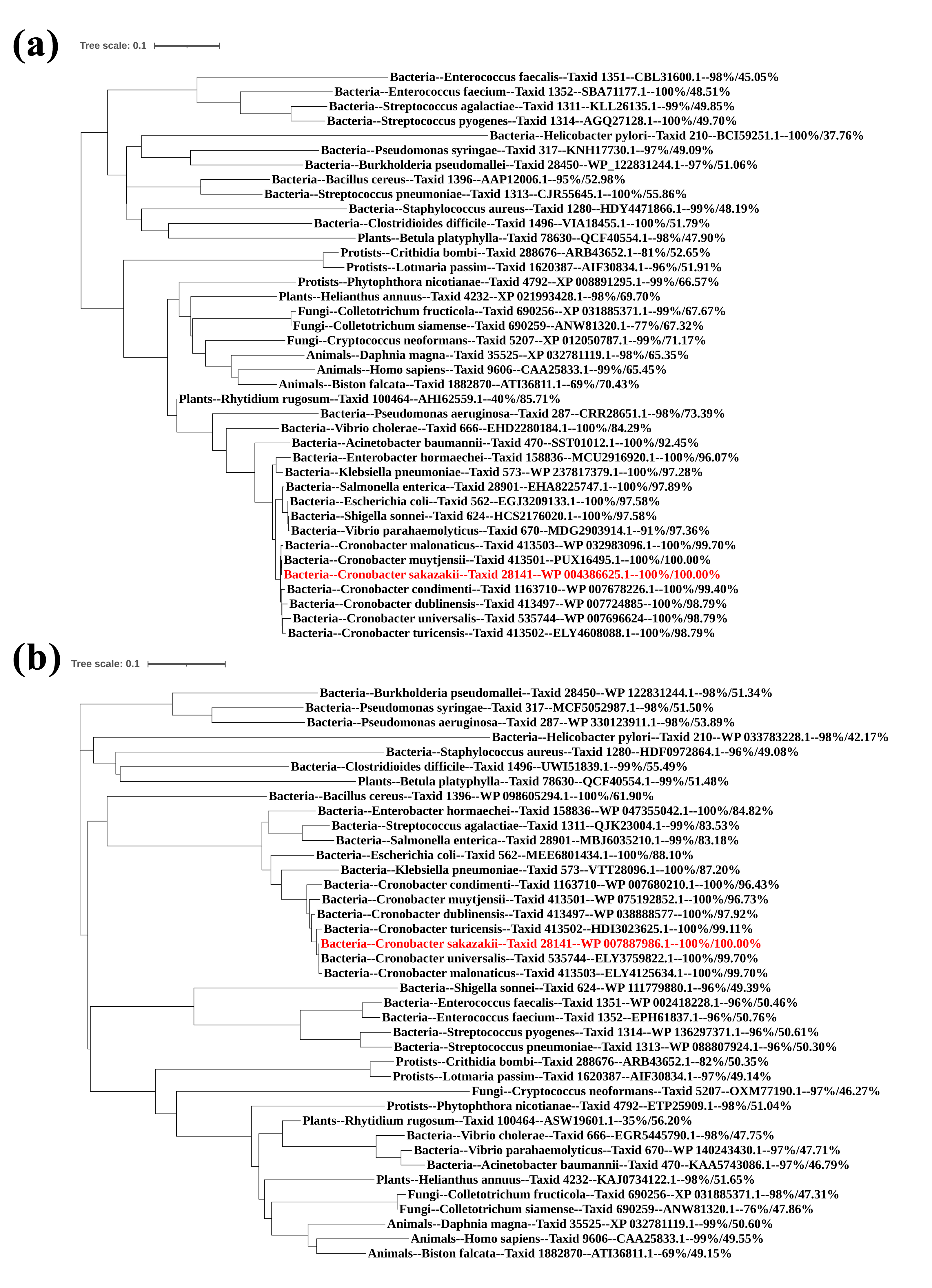

Supplement: Figure S1.jpg [file KVIR_A_2446713_SM3657.jpg]

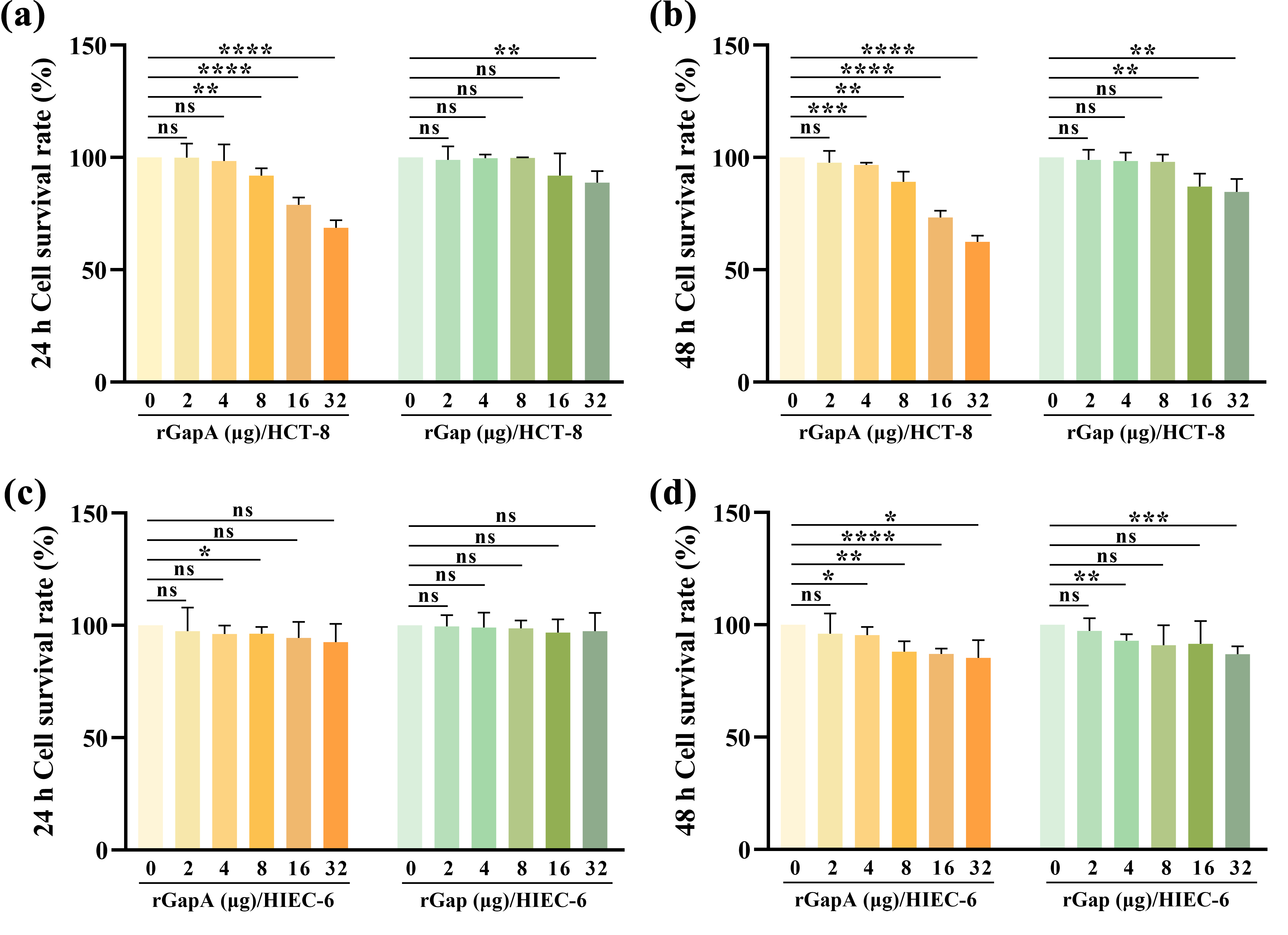

Supplement: Figure S3.jpg [file KVIR_A_2446713_SM3656.jpg]

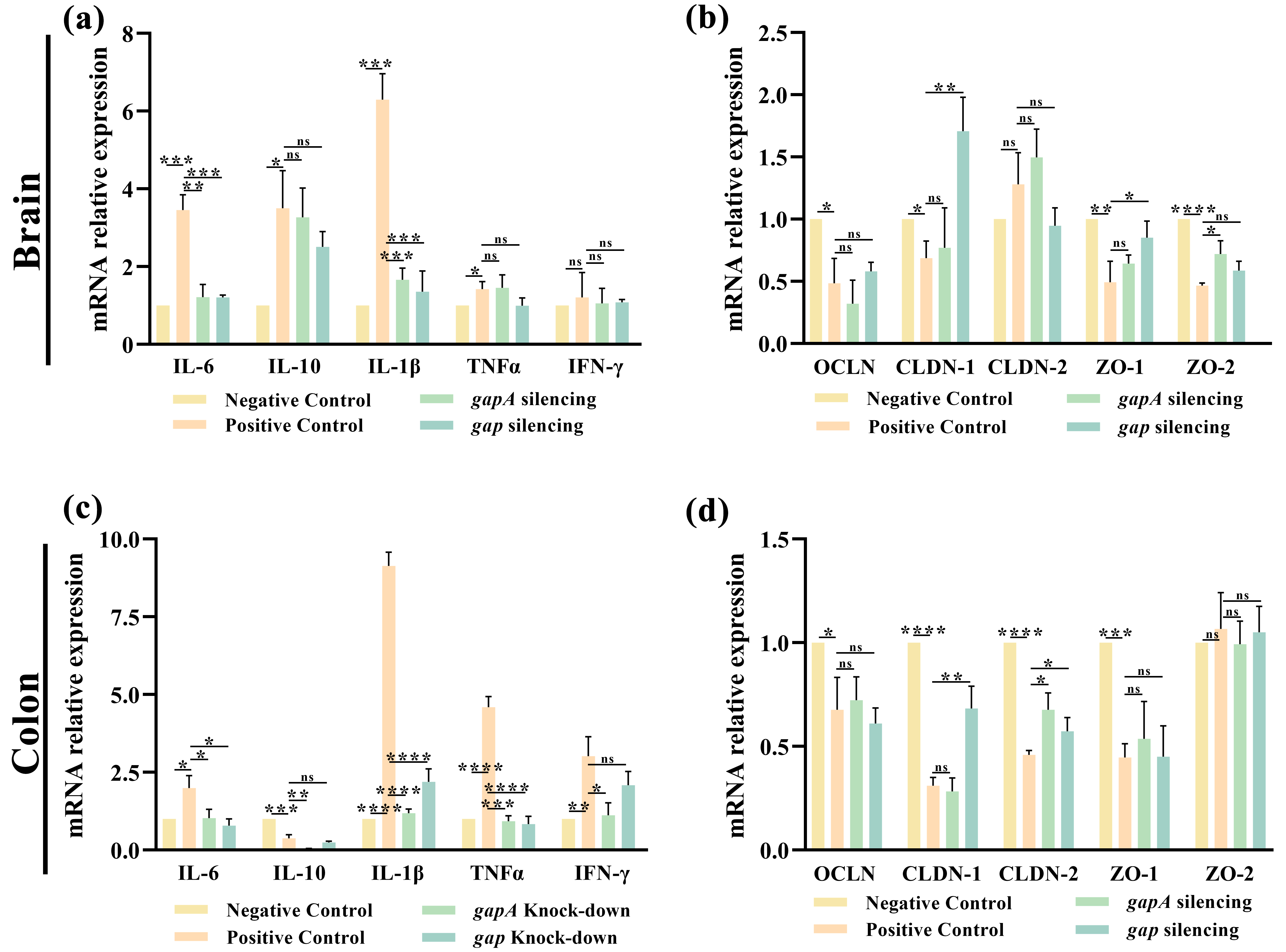

Supplement: Figure S5.jpg [file KVIR_A_2446713_SM3655.jpg]

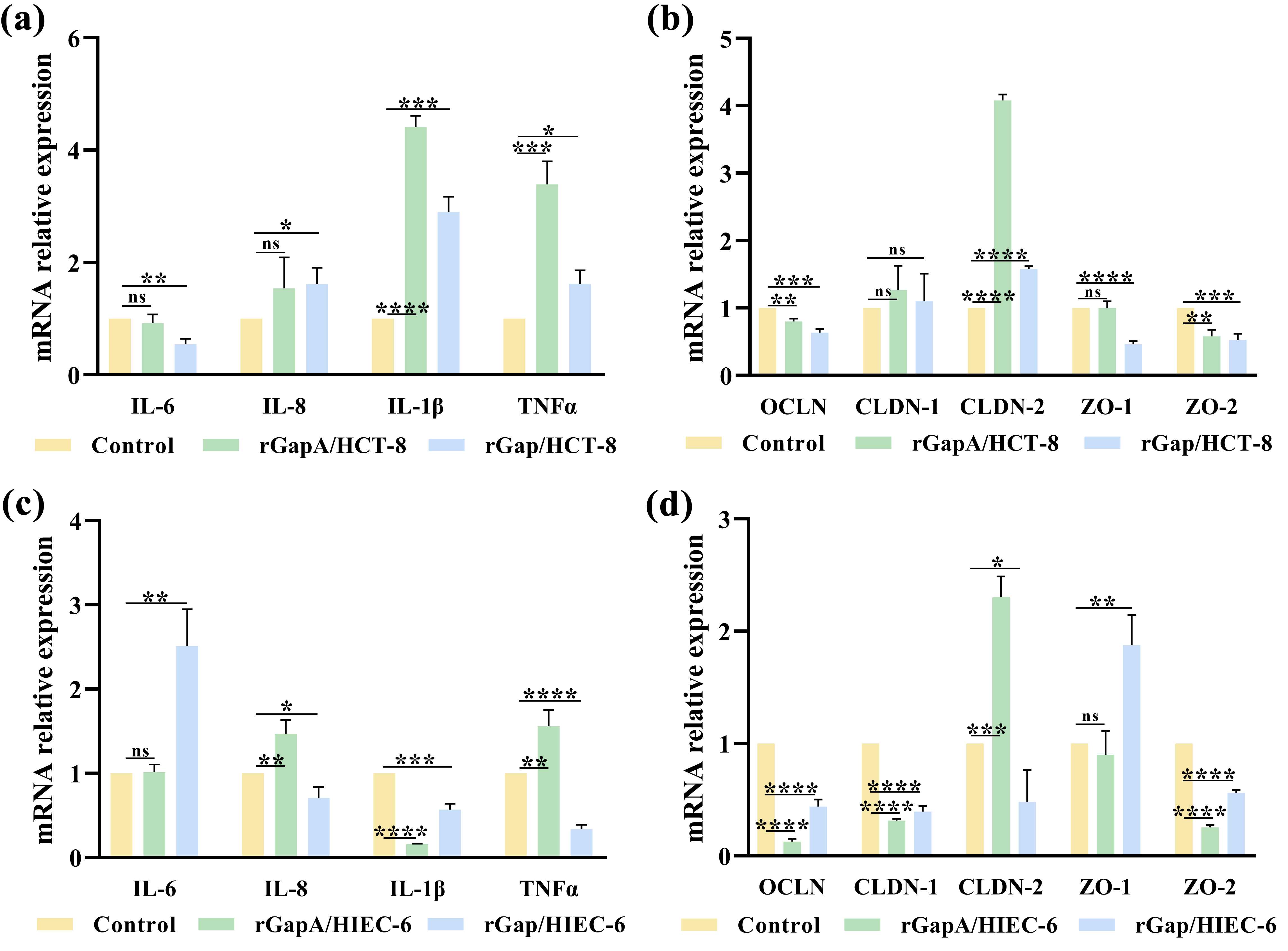

Supplement: Figure S4.jpg [file KVIR_A_2446713_SM3653.jpg]
